# A New Extremotolerant Ecotype of the Fungus *Pseudotaeniolina globosa* Isolated from Djoser Pyramid, Memphis Necropolis, Egypt

**DOI:** 10.3390/jof7020104

**Published:** 2021-02-02

**Authors:** Samah Mohamed Rizk, Mahmoud Magdy, Filomena De Leo, Olaf Werner, Mohamed Abdel-Salam Rashed, Rosa Maria Ros, Clara Urzì

**Affiliations:** 1 Department of Plant Biology, Faculty of Biology, Murcia University, 30100 Murcia, Spain; werner@um.es (O.W.); rmros@um.es (R.M.R.); 2 Genetics Department, Faculty of Agriculture, Ain Shams University, Cairo 11241, Egypt; m.elmosallamy@agr.asu.edu.eg (M.M.); rashed5012@agr.asu.edu.eg (M.A.-S.R.); 3 Department of Chemical, Biological, Pharmaceutical and Environmental Sciences, University of Messina, 98166 Messina, Italy; fdeleo@unime.it (F.D.L.); urzicl@unime.it (C.U.)

**Keywords:** black yeasts, Capnodiales, Dothideomycetes, halotolerant fungi, multi-locus genotyping, rock-inhabiting fungi, Teratosphaeriaceae

## Abstract

Most of the rock-inhabiting fungi are meristematic and melanized microorganisms often associated with monument biodeterioration. In previous microbial profiling of the Egyptian Djoser pyramid, a *Pseudotaeniolina globosa* isolate was found. The current study aimed to characterize the *P. globosa* isolated from the Djoser pyramid compared with an Italian isolate at morphological, physiological, and molecular levels. Experiments were carried out to test temperature, salinity, and pH preferences, as well as stress tolerance to UV radiation and high temperature, in addition to a multi-locus genotyping using ITS, nrSSU or 18S, nrLSU or 28S, BT2, and RPB2 markers. Morphological and molecular data confirmed the con-specificity of the two isolates. However, the Egyptian isolate showed a wider range of growth at different environmental conditions being much more tolerant to a wider range of temperature (4–37 °C) and pH values (3.0–9.0 pH) than the Italian (10–30 °C, 4.0–6.0 pH), and more tolerant to extreme salinity levels (5 M NaCl), compared to the lowest in the Italian isolate (0.2 M NaCl). Besides, the Egyptian isolate was more tolerant to high temperature than the Italian isolate since it was able to survive after exposure to up to 85 °C for 5 min, and was not affected for up to 9 h of UV exposure, while the Italian one could not regrow after the same treatments. The *Pseudotaeniolina globosa* species was attributed to the family Teratosphaeriaceae of the order Capnodiales, class Dothideomycetes. Our results demonstrated that the Egyptian isolate could be considered an ecotype well adapted to harsh and extreme environments. Its potential bio-deteriorating effect on such an important cultural heritage requires special attention to design and conservation plans and solutions to limit its presence and extension in the studied pyramid and surrounding archaeological sites.

## 1. Introduction

Black meristematic fungi or micro-colonial fungi [1,2], which were more recently referred to as “rock-inhabiting fungi” (RIF) [3], are slow-growing microorganisms often associated with natural rocky substrates [4,5]. To economize the energy requirements in extreme environments, their life cycles are simplified [6], lacking morphologically differentiated sexual phases, and producing only a few metabolites and morphological structures crucial for survival [7]. This group of fungi possesses peculiar characters related to stress tolerance (e.g., melanized cell walls), enabling them to successfully reside under harsh climatic conditions of prolonged desiccation, extreme temperatures (even in Arctic habitats), high solar irradiation and osmotic stress, and limited nutrient availability [6,8], where cosmopolitan and fast-growing micro-fungi are unable to survive [5]. Although the role of black meristematic fungi in monument decay remained underestimated for a long time, it is now clear that they are among the most active groups of microorganisms causing weathering of rocks and biodeterioration of monuments exposed to outdoor conditions [9,10,11,12,13,14,15] in addition to their consistent detection on marble monuments in the Mediterranean basin [16,17,18,19,20].

The taxonomic diversity of RIF appears to be unexpectedly wide, although only a limited number of RIF species and genera have been described [21]. Several studies have revealed that the ability of micro-fungi to grow on rocky substrates is a polyphyletic trait, assessing RIF in two different classes of Ascomycota, namely Dothideomycetes (mainly the orders Capnodiales, Dothideales, and Pleosporales) and Eurotiomycetes (order Chaetothyriales) [3,22,23,24,25,26]. Their identification also remained relatively intuitive because of their scarce differentiation and morphological plasticity, until molecular techniques became common in fungal systematics. With the improvement of isolation procedures and molecular methods, it has become apparent that RIFs are much more common and widespread than previously believed [3].

Within the RIF group, *Pseudotaeniolina* J.L. Crane & Schokn. (order Capnodiales) was introduced as a monotypic genus for a species isolated from plant material named *P. convolvuli* (Esfand.) J.L. Crane & Schokn. De Leo et al. [16] isolated the second species, *P. globosa* De Leo, Urziì & De Hoog, from the outside wall of the church of ‘‘Santa Maria di Mili’’ in Messina, Italy. These authors described it as an anamorphic, melanized fungus with meristematic development followed by arthric secession leading preponderantly to single cell formation. The identification was supported by SSU (small subunit) and ITS (internal transcribed spacer) rDNA sequence data. However, the family name was still not assigned [27]. Currently, *P. globosa* has few isolates worldwide and a limited dataset available in the GenBank database (https://www.ncbi.nlm.nih.gov/genbank/, accessed August, 2020). 

Physical, chemical, and biological factors play a combined role in weathering archaeological sites suffering from biodeterioration. Previously, in Egyptian monuments, species from genera *Alternaria* Nees, *Aspergillus* P. Micheli ex Haller, *Bipolaris* Shoemaker, *Cladosporium* Link, *Dichotomopilus* X. Wei Wang, Samson & Crous, *Fusarium* Link, *Rhizopus* Ehrenb. and *Penicillium* Link were reported from several archaeological remains. Among these are the archaeological tombs in Alexandria city [28], Tuna el-Gabel’s excavations near Al-Minya city [29], the Great pyramid complex (Giza city), the Mosque of judge Abd El basset (Cairo city), the Museum of Ismailia Antiquities (Ismailia city), the National Museum of Egyptian Civilization (Cairo city), Senusret Ι obelisk (Fayoum city), and Seti Ι tomb (Abydos city) as described by Mohamed & Ibrahim [30]. Besides, a microbial survey performed in the Djoser pyramid complex using metabarcoding and traditional isolation methods found *Pseudotaeniolina globosa* on Egyptian archaeological remains (unpublished), this being the second worldwide report of the species after its description in Messina by De Leo et al. [16].

This study aimed to deepen the previous finding and had the following specific objectives: (1) to evaluate if the sample of *P. globosa* isolated from the step pyramid of Djoser corresponds to the same isolate found previously in Messina or is perhaps an extremotolerant ecotype; (2) in an affirmative case, to describe this ecotype, characterizing it at morphological, physiological and molecular levels and knowing its UV and temperature tolerance; (3) to better clarify the taxonomic position within the order Capnodiales based on multi-locus genotyping identification. 

## 2. Materials and Methods

### 2.1. Isolate Sampling

The Pyramid of Djoser, also known as the “Step Pyramid”, is an archaeological remain in the Saqqara region in the Memphis necropolis, located in the northern part of the Nile Valley, situated at 29°52′10.17″ N and 31°13′8.70″ E in the Giza governorate, Egypt. The *Pseudotaeniolina globosa* isolate used in the current study, coded as DPS10, was obtained from soil particles collected at the Djoser pyramid’s ground entrance using Dichloran Rose Bengal Chloramphenicol (DRBC) agar medium (#CM0727, Oxoid, Ontario, Canada). The nomenclature of fungi follows Index Fungorum (http://www.indexfungorum.org).

### 2.2. Morphological Characterization of Pseudotaeniolina Globosa

Hyphal maturation and conidiogenesis were observed. Culture media preference experiments were also performed.

#### 2.2.1. Hyphal Maturation and Conidiogenesis

These were studied using light and phase contrast microscopy on slide cultures performed by inoculating the DPS10 in squared agar blocks of Malt Extract Agar (MEA; #1038, Condalab, Madrid, Spain). The slide cultures were incubated for one month at 25 °C in wet sterile Petri dishes with filter paper to avoid media dehydrating and observed using lactophenol by a Leica DMLB Tilting Trinocular Phase Contrast (100×) and Dark Field Light Microscope (100×). Digital images were captured using a Leica DFC500 digital color camera optimized with the software Micromax Arkon (v. 8.12.05).

#### 2.2.2. Culture Media Preferences

Characteristics and growth rates of the colonies were studied and recorded after one month, performed in duplicates of divided Petri dishes of four sections, which contained MEA, Potato Dextrose Agar (PDA; #1022, Condalab, Spain), Oatmeal Agar (OMA; #2060, Condalab, Spain), and Czapek Dox Agar (CzA; #1015, Condalab, Spain) incubated at 25 °C.

### 2.3. Physiological Characterization of Pseudotaeniolina Globosa

Temperature preferences, growth at different salt concentrations, and growth at different pH were performed according to Selbmann et al. [8]. Colonies with diameter >2 mm were considered positive, according to Kane & Summerbell [31]. Comparisons with an Italian isolate (MC769) of the species were made based on the data reported by De Leo et al. [16].

#### 2.3.1. Temperature Preferences

PDA Petri dishes divided into four sections in duplicates containing the DPS10 isolate were incubated at 4, 10, 25, 30, 34, and 37 °C. The colony diameter was recorded after one month of incubation to detect the isolate’s optimum growth among the different selected temperatures.

#### 2.3.2. Growth at Different Salt Concentrations

An experiment was performed to test the ability of the fungus to grow in the presence of a different concentration of salts and to recognize its salinity preference by inoculating the DPS10 isolate on four spots in duplicates of MEA plates supplemented with a scale of NaCl concentrations of 1.2, 1.5, 3.0, 5.0, 7.0, 10.0, 12.0, 15.0, 18.0, 25.0, and 30.0%, incubated at 25 °C for one month.

#### 2.3.3. Growth at Different pH

The DPS10 isolate capacity to grow at different pH values was tested in triplicates of MEB medium at pH levels of 1.0, 2.0, 3.0, 4.0, 5.0, 6.0, 7.0, 8.0, and 9.0. At standard conditions for RIFs pH growth [8], the experiment was initially performed at pH 5.0 by 1N HCl; then media was adjusted for pH 1.0 using HCl/KCl buffer and for pH 2.0–7.0 by using McIlvaine solution and for pH 8.0–9.0 by applying Clark & Lubs solution. DPS10 isolate was incubated at 25 °C for one month in a shaken culture at 70 rpm [32].

### 2.4. Tolerance Assessment of Pseudotaeniolina Globosa

As *P. globosa* is exposed to the sun and dryness prevailing in the Djoser pyramid area, it is supposed to be adapted to this arid environment. In addition to the DPS10, isolate MC769 of *P. globosa* (CBS 109889T) [16] was used for tolerance assessment and molecular analyses. The MC769 was obtained from the fungal collection of the Department of Chemical, Biological, Pharmaceutical and Environmental Sciences, University of Messina, Italy, and freshly re-cultivated using the culture medium Malt Extract Broth (MEB; #CM0057, Oxoid, USA). Experiments were designed to recognize the extreme UV radiation levels and temperature that the DPS10 and MC769 isolates can tolerate.

#### 2.4.1. UV Tolerance under Wet and Dry Conditions

The ability of the fungus to survive and reproduce after exposure to germicide UV-C radiation (253.7 nm) in wet and dry conditions was tested. The experiment material was prepared in replicates by placing about 1 cm^2^ mycelia of one month old cultures of DPS10 and MC769 isolates in an open sterile Petri dish each along with 1 mL physiological solution (0.9% NaCl), where the solution was not more than 1 mm deep (wet condition), or without the physiological solution (dry condition). The Petri dishes were then placed at 35 cm from the UV lamp (Philips TUV T8 30W). The sampling was done in duplicates after 10 and 30 min, 1, 3, 6, 9, 12, and 24 h. The treated mycelia under wet and dry conditions were re-planted in PDA medium and incubated at 25 °C for one month to record their regrow ability.

#### 2.4.2. Heat Stress Tolerance

The experimental aim was to identify the degree of the fungus ability to survive and resume growth after the exposure of the cells to high-temperature stress. The experiment was performed in duplicates by harvesting about 1 cm^2^ mycelia of one month old cultures of DPS10 and MC769 isolates and transfer them to 1 mL of physiological solution in 2 mL tubes. The tubes were put in a water bath adjusted to 65 °C up to 85 °C in 5 °C intervals. The sampling was done in duplicates after 2, 5, 10, and 15 min of exposure at each temperature. The same was repeated in dry conditions using hot blocks without a physiological solution. The treated mycelia from both wet and dry conditions were re-planted in PDA medium and incubated at 25 °C for one month.

### 2.5. Molecular Characterization of Pseudotaeniolina Globosa

Molecular characterization of both isolates and family assignment of *P. globosa* were performed using a PCR based approach combined with Sanger sequencing, commonly known as “multi-locus genotyping” using five molecular markers.

#### 2.5.1. DNA Extraction

DNA for both fungal isolates was extracted using cetyltrimethylammonium bromide (CTAB) acid-washed beads using the manual protocol of Möller et al. [33] and modified by Urzì et al. [34]. DNA was quantified with Qubit^TM^ and the Qubit^TM^ dsDNA BR assay kit (Invitrogen, Thermo Fisher Scientific, Massachusetts, MA, USA).

#### 2.5.2. Multi-Locus Genotyping

The loci that were amplified for multi-locus comparison between the two isolates were the ITS, the small subunit of the nuclear ribosomal RNA (nrSSU or 18S), the large subunit of the nuclear ribosomal RNA (nrLSU or 28S), B-tubulin (BT2), and RNA polymerase II (RPB2) [21]. Primers are listed in Table 1.

PCR reactions were performed using the MyTaq™ Red Mix (Cat# BIO-25043, BioLine, London, UK). Each 25 µL reaction tube included 5 pmol of each primer, and 40 ng of template DNA. The amplification was carried out using a Techne™ 512 thermocycler (Techne, Staffordshire, UK). The PCR programs were adjusted according to the primer pair melting temperature (Tm) as follows: the first denaturation step at 95 °C for 5 min was followed by 33 cycles of denaturation at 95 °C for 30 s, annealing at the assigned temperature (see Table 1) for 30 s, extension at 72 °C for 30 s and a final extension 72 °C for 5 min.

PCR products were visualized using 1.5% agarose gel electrophoresis in 1x TBE buffer. When successful, all PCR reactions were prepared for the cleanup step (purification) using GeneJET^TM^ PCR purification kit (K0702, Fermentas, Thermo Fisher Scientific, Massachusetts, MA, USA) before automated Sanger sequencing (ABI 3730xl System, Macrogen, Inc., Seoul, Korea). Chromatographs of both directions were trimmed, assembled, and aligned using Geneious Prime [35] and blasted for species identification using the3 NCBI online Blast tool (https://blast.ncbi.nlm.nih.gov/Blast.cgi). Retrieved sequences were revised and checked using GenBank nucleotide database (https://www.ncbi.nlm.nih.gov/nuccore).

Taxonomic ranking and phylogenetic relationships were retrieved from the NCBI taxonomy database (https://www.ncbi.nlm.nih.gov/taxonomy). Alignments of the target sequences and the BLAST query results were performed using ClustalW [36] using the default settings, while repeated sequences were discarded. Phylogenetic trees were constructed by the maximum likelihood method based on the Generalized Time-reversible (GTR) model using Fasttree v2.1.5 [37].

## 3. Results

### 3.1. Morphological Characterization of Pseudotaeniolina Globosa

#### 3.1.1. Hyphal Maturation and Conidiogenesis

**Figure 1:**
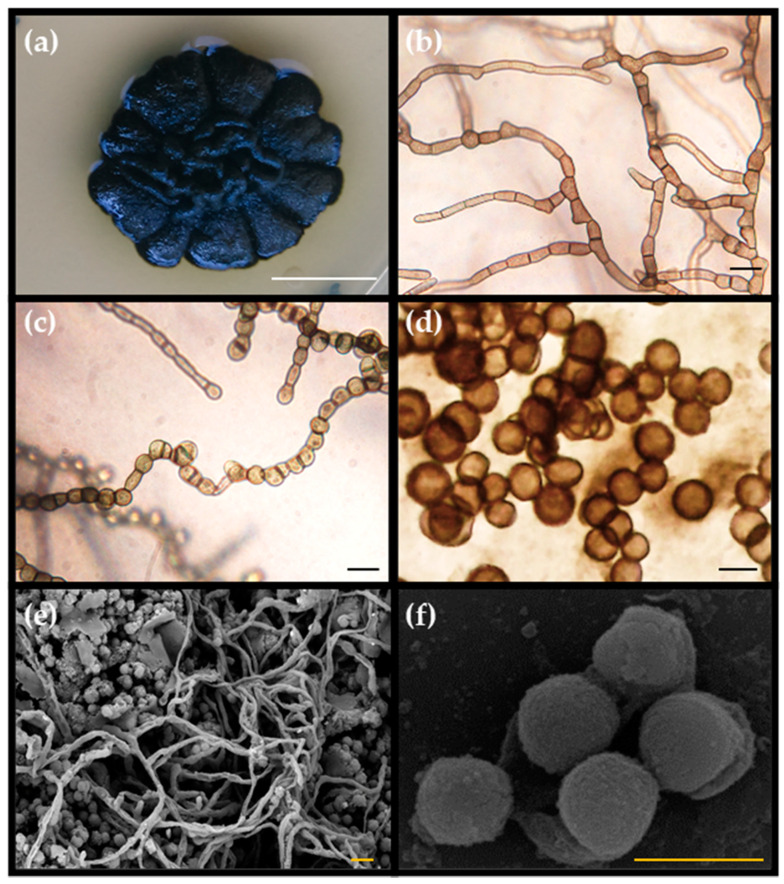
*Pseudotaeniolina globosa* DPS10 isolate morphology. (**a**) Colony after one-month incubation on Malt Extract Agar (MEA) medium; (**b**) Developing hyphae; (**c**) Clumps of meristematic structure; (**d**) Mature spherical conidia; (**e**) Hyphal structure and mature conidia; (**f**) Mature conidia. (**a**–**d**) Micrographs under the light microscope. (**e**,**f**) Micrographs under the Scanning Electron Microscope. White bar = 1 cm, black bars = 20 µm, and orange bars = 10 µm.

The DPS10 isolate was anamorphic. Colonies grew slowly as they reached 21.0 ± 0.8 mm in diameter after one month. Yeast-like cell forms were absent, and the teleomorph was not observed. Black colonies were butter-like (butyreae); they became shiny and of more rigid structure and wrinkled, with a cauliflower shape, with age. The mycelium was composed of pale brown, thick-walled immersed, branched hyphal cells, 7.0–12.0 µm long, and 4.0–6.0 µm wide. The meristematic mycelium eventually converted into multicellular clumps, 5.0–12.0 µm in diameter. Conidia were produced by arthric disarticulation of hyphae; these were uni- or bi-cellular, pale brown, constricted at the septa, and 4.8–5.5 µm in diameter (Figure 1). 

#### 3.1.2. Culture Media Preferences

**Figure 2:**
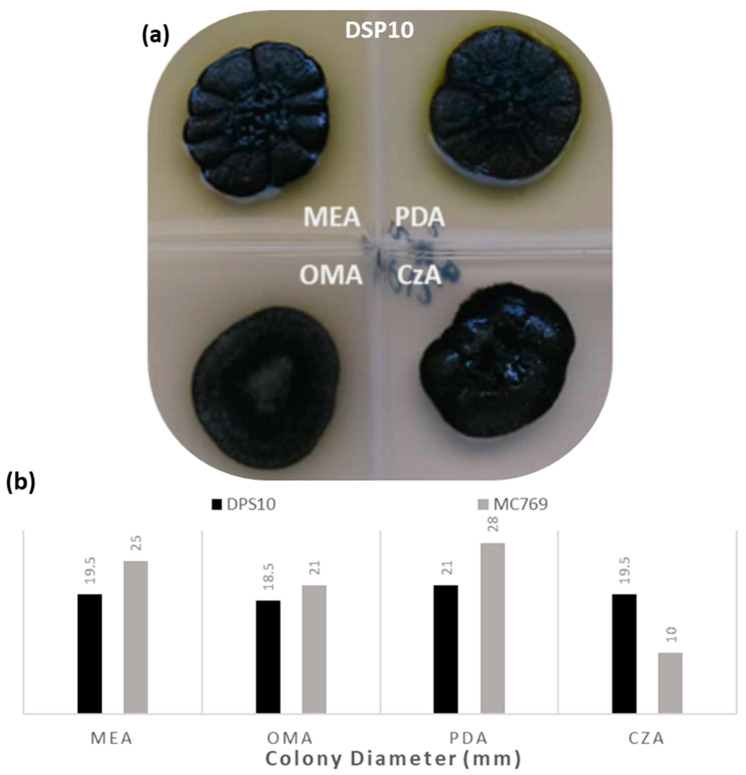
Colonies of *Pseudotaeniolina globosa* grown on different media (MEA, Oatmeal Agar (OMA), Potato Dextrose Agar (PDA), Czapek Dox Agar (CzA)). (**a**) Colonies of DPS10 isolate on the four media; (**b**) Colonies diameter are compared with MC769 isolate according to published data by De Leo et al. [16].

The isolate DPS10 was able to grow and reproduce on different culture media after one month of incubation at 25 °C. On PDA, it showed a visible optimum growth with a diameter record of 21.0 ± 0.8 mm, with black, shiny, hard, wrinkled structure and a cauliflower shape, slightly raised at the center (umbonate), radially folded, with a unique appearance of an olive-green glow surrounding the colony edge. On MEA and CzA media, it showed a small reduction of 19.5 ± 0.7 mm in diameter, while the same morphological appearance was observed, except an olive-green color on MEA, and change from umbonate to crateriform with leathery texture on CzA. The smallest ultimate colony diameters were observed on the OMA medium, being 18.5 ± 0.8 mm in diameter, with a different appearance of a flat, hairy, gray growth on the colony top. No changes in the conidia morph among the tested media were observed (Figure 2). 

As reported by De Leo et al. [16], MC769 isolate colony on PDA showed the highest growth record of 28.0 mm in diameter with flat, regular margin, slightly raised at the center, cerebriform, and radially folded. On MEA, the colony was black and shiny, buttery, flat, slightly raised at the center and of 25.0 mm in diameter. It had a sharp, regular margin of 21.0 mm diameter on OMA, while the colony on CzA was flat, with a fimbriate margin, attaining up to 10.0 mm diameter (Figure 2).

### 3.2. Physiological Characterization of Pseudotaeniolina Globosa

#### 3.2.1. Temperature Preferences

**Figure 3:**
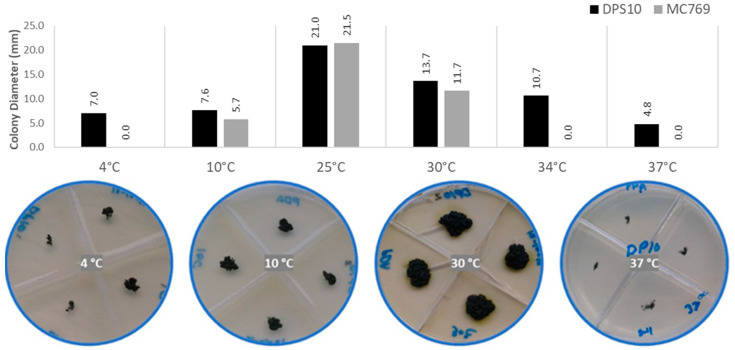
Temperature preferences of *Pseudotaeniolina globosa* DPS10 isolate shown by the colony diameter reached on PDA medium after one-month incubation. The column chart in the upper part exhibit comparison of DPS10 and MC769 isolates at 4, 10, 25, 30, 34, and 37 °C. Visual examination of the colonies in the lower part shows the growth of DPS10 at 4, 10, 30, and 37 °C in quadruplicates (Petri dish of four divisions). Data on MC769 isolate come from De Leo et al. [16].

The DPS10 isolate grew and reproduced at a wide range of temperatures, ranging from 4 to 37 °C, with the optimum growth observed at 25 °C where the colony reached 21.0 ± 0.7 mm in diameter. Above this temperature, a considerable reduction of colony diameter occurred at 30 °C with 13.7 ± 0.3 mm, and 37 °C with 4.8 ± 0.4 mm in diameter. At the lowest temperature at which the colony was able to grow, it reached 7.0 ± 0.7 mm in diameter. Considering these data, it can be referred to as a mesophilic-psychrotolerant fungus. In comparison, as De Leo et al. [16] reported, the isolate MC769 showed the same diameter of optimum growth at 25 °C and near same diameter, between 10 to 30 °C, without any record of growth at lower and higher temperature degrees (Figure 3).

#### 3.2.2. Growth at Different NaCl Concentrations

**Figure 4:**
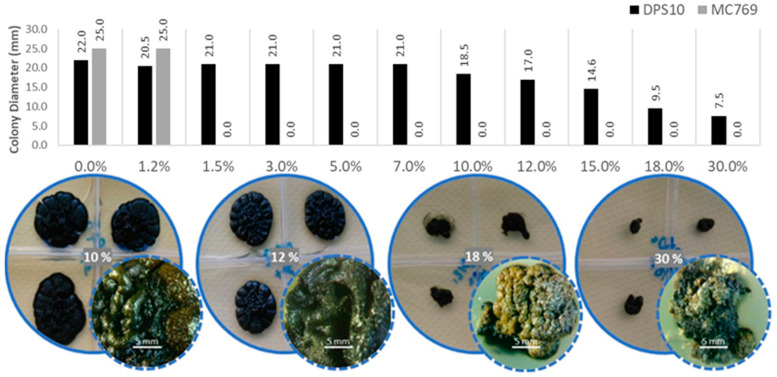
Salinity preferences of *Pseudotaeniolina globosa* DPS10 isolate shown by the colony diameter reached on MEA medium after one-month incubation at 25 °C. Column chart in the upper part exhibit comparison of DPS10 and MC769 isolates in medium supplemented with 0.0, 1.2, 1.5, 3.0, 5.0, 7.0, 10.0, 12.0, 15.0, 18.0 and 30.0% NaCl. Visual examination of the cultured plates in the lower part shows the growth of DPS10 isolate at 10.0, 12.0, 18.0, and 30.0% NaCl in quadruplicates (Petri dish of four divisions) with a stereomicroscopic focus for one of the grown colonies (bar = 5 mm). Data on MC769 isolate come from De Leo et al. [16].

The DPS10 isolate could grow at all the tested NaCl concentrations, even at 30.0%, the maximum checked. It showed colonies of 22.0 ± 0.5 to 18.5 ± 1.2 mm diameter up to 10.0% of NaCl concentration and showed a limited reduction of colony diameter of 17.0 ± 1.9 and 14.6 ± 1.6 mm at 12.0% and 15.0% NaCl concentration, respectively; and a considerable reduction to 9.5 ± 1.7 and 7.5 ± 0.8 mm at 18.0% and 30.0% NaCl concentration, respectively. Differently, MC769 isolate only grew at a maximum of 1.2% NaCl concentration as reported by De Leo et al. [16] (Figure 4).

#### 3.2.3. Growth at Different pH

**Figure 5:**
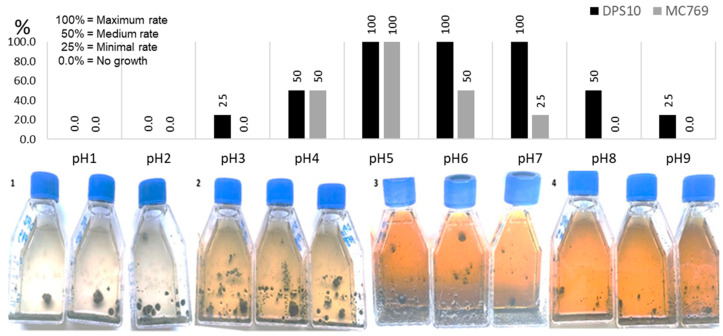
The pH growth range of *Pseudotaeniolina globosa* DPS10 isolate shown by four established growth grades (0.0, 25%, 50%, 100%) reached on Malt Extract Broth (MEB) medium after one-month incubation at 25 °C. Column chart in the upper part exhibits comparison of DPS10 and MC769 isolates measured at pH 1.0 to pH 9.0. Flasks in the lower part show growth at pH 4.0 (1), pH 6.0 (2), pH 8.0 (3), and pH 9.0 (4). Data on MC769 isolate come from De Leo et al. [16].

The DPS10 isolate showed an ability to grow at a wide range of pH values (from pH 3.0 to pH 9.0). The maximal growth was recorded between pH 5.0 and pH 7.0, and the minimal growth was recorded at pH 3.0 and pH 9.0. Based on the data from De Leo et al. [16], MC769 isolate showed growth at a narrower range of pH values (from pH 4.0 to pH 7.0), with maximal growth at pH 5.0 and minimal growth at pH 7.0. Comparing the two isolates, both started to grow at pH 4.0 with optimal growth up to pH 5.0 for MC769 and up to pH 7.0 for DPS10 and continued with growth reduction to pH 9.0. On the contrary, MC769 showed no growth at pH 8.0 (Figure 5).

### 3.3. Tolerance Experiments of Pseudotaeniolina Globosa

#### 3.3.1. UV Tolerance under Wet and Dry Conditions

**Figure 6:**
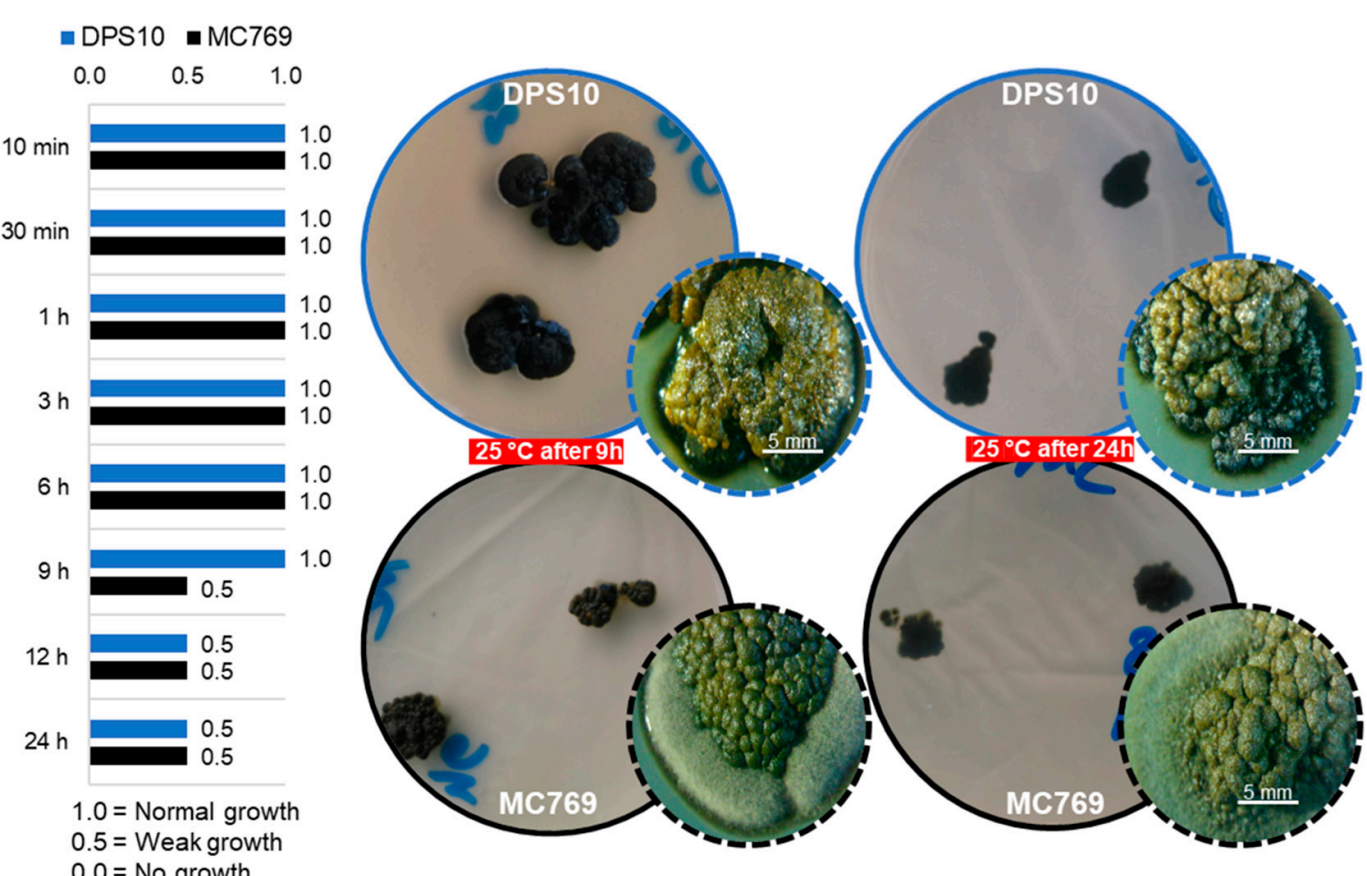
UV tolerance examination of *Pseudotaeniolina globosa* DPS10 and MC769 isolates. Column chart on the left side exhibits comparison of DPS10 and MC769 isolates growth monitored on PDA media at 25 °C after 10 and 30 min, 1, 3, 6, 9, 12, and 24 h of exposure shown by three established growth grades (0.0, 0.5, 1.0). Visual examination of the cultured plates on the right side shows the growth of DPS10 and MC769 isolates after 9 and 24 h, with a stereomicroscopic focus for one of the grown colonies (bar = 5 mm).

The isolates DPS10 and MC769 showed an equal maximum growth rate after UV exposure of 10 min until 6 h; the isolates maintained the same colony diameter (21.0 and 25.0 mm, respectively). MC769 showed a reduction of 50% of average colony diameter (after 9, 12, and 24 h of UV exposure), while DPS10 showed 50% reduction after 12 and 24 h of UV exposure (Figure 6).

#### 3.3.2. Heat Stress Tolerance

**Figure 7:**
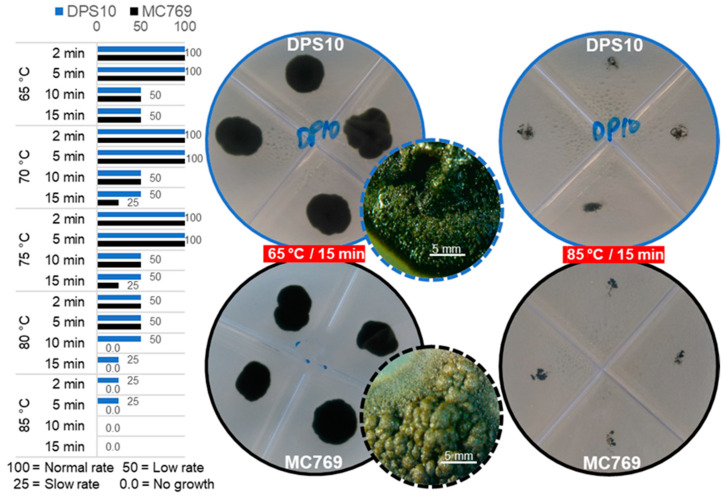
Heat stress tolerance examination of *Pseudotaeniolina globosa* DPS10 and MC769 isolates. Column chart on the left exhibits comparison of DPS10 and MC769 isolates growth monitored on PDA media after 2, 5, 10, and 15 min at 65, 70, 75, 80, and 85 °C; the x-axis represents the grade from 100 for regular growth rate after one month of incubation (21.0 and 25.0 mm colony diameter for DSP10 and MC769, respectively) to 0.0 for no growth. Visual examination of the cultured plates on the right shows the growth of DPS10 and MC769 at 65 and 85 °C after 15 min of heat exposure, with a stereomicroscopic focus for one of the grown colonies (bar = 5 mm).

Heat stress on isolates DPS10 and MC769 at 65, 70, and 75 °C after 2 and 5 min showed the maximum growth rate a colony can reach in one month (colony diameter of 21.0 and 25.0 mm, respectively). However, when the exposure extended to 10 and 15 min, a lower growth rate was observed at all the tested temperatures for both isolates. At 70 and 75 °C, after 15 min of exposure, the MC769 showed a slower rate than the low rate described for DPS10. When the 80 °C was tested, both isolates were grown at a low rate after 2 and 5 min of exposure; however, only DPS10 maintained the same rate after 10 min and showed a slow rate after 15 min of exposure contrast to MC769, which showed no growth. When the 85 °C was tested, only DPS10 grew at a slow rate after 2 and 5 min, while showed no growth after 10 and 15 min of exposure (Figure 7).

### 3.4. Molecular Characterization and Phylogeny of Pseudotaeniolina Globosa

The amplification of the five loci for the two isolates was successful, showing the expected molecular size as previously reported. Sequences were deposited in GenBank repository under the accession numbers detailed in Table 2. Based on the BLAST results, similar top results were retrieved and filtered by query coverage (i.e., <95% query coverage were discarded) and matched with “Popsets” files downloaded from the NCBI database for each locus prior to alignment and phylogenetic analysis. All the retained accessions belonged to the order Capnodiales. The maximum likelihood (ML) phylogenetic trees were rooted with the Extremaceae family species, while all the remaining sequences belonged to the families Teratosphaeriaceae and Mycosphaerellaceae for all the genotyped loci. In the case of ITS, a total of 30 accessions belonging to the order Capnodiales in addition to the current two isolates of *P. globosa* (DPS10 and MC769) showed an alignment length of 566 bp, including partial 5′ and 3′ ends of both ITS1 and ITS2, respectively, and the complete 5.8S locus. 

**Figure 8:**
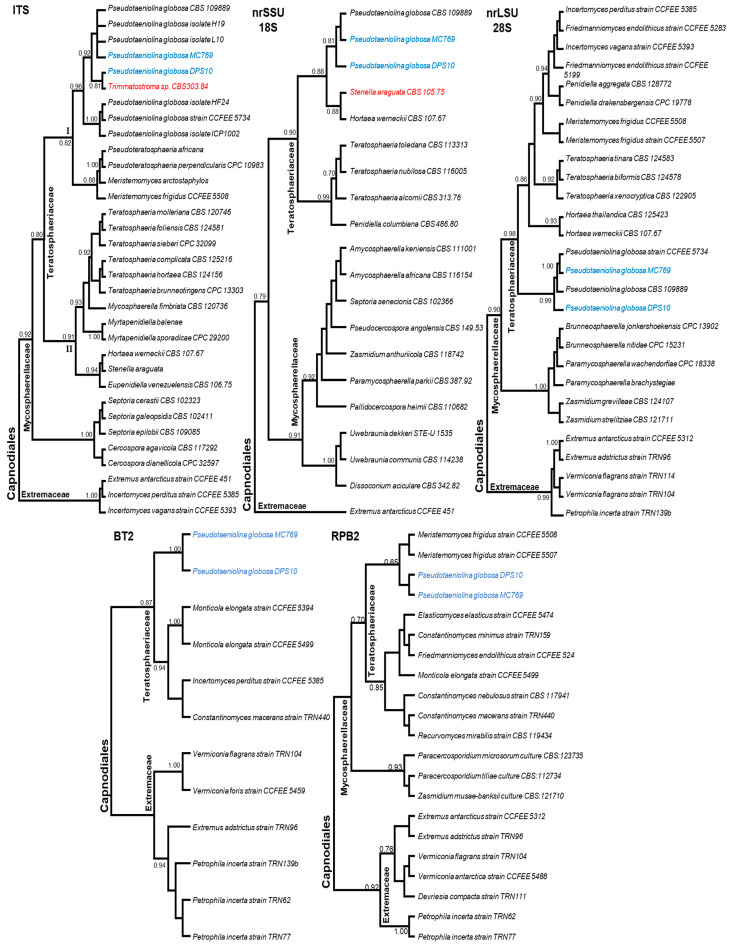
Maximum likelihood-based phylogenetic trees for top similar accessions of Extremaceae, Mycosphaerellaceae, and Teratosphaeriaceae families (order Capnodiales) from GenBank NCBI database to ITS, nrSSU 18S, nrLSU 28S, BT2 and RPB2 sequences of *Pseudotaeniolina globosa* isolates (MC769 and DPS10 written in blue color). Trees are rooted by the Extremaceae clade/species; mistakenly labeled/grouped accessions are written in red.

The number of identical sites was 253 bp (50%), while the un-gapped average length was 446 bp with GC% = 55.9%. A major observation is that the family Teratosphaeriaceae members were highly clustered into two sub-clades (I and II; bootstrap value ≥ 0.80). The isolate *P. globosa* DPS10 was highly clustered to eight *P. globosa* accessions (bootstrap value = 0.96). The most similar sequence to the Egyptian isolate (with bootstrap value = 0.81) was the specimen CBS303.84, which was early registered in GenBank as *Trimmatostroma* sp. [44] and corrected to *P. globosa* in a later publication [8]. The geolocation of the samples (Table 2) does not reflect the phylogenetic relationships based on this marker (Figure 8, ITS). 

In the case of the nrSSU (18S), a total of 18 accessions belonging to the order Capnodiales in addition to the current two isolates of *P. globosa* (DPS10 and MC769) showed an alignment length of 1670 bp. The number of identical sites was 1541 bp (92.3%), while the un-gapped average length was 1667 bp with GC% = 48.5%. The *P. globosa* isolates were included within the clades identified as order Capnodiales. In addition to DPS10 and MC769 isolates, a single *P. globosa* accession CCFEE 5734 was highly clustered in the same clade (bootstrap value = 0.92). The *P. globosa* accessions were sister to *Hortaea werneckii* (Horta) Nishim. & Miyaji (CBS 107.67), a very peculiar species known as an extremophilic halotolerant fungus, and *Stenella araguata* Syd. (CBS 105.75) (family Mycosphaerellaceae) a biological causal agent for Tinea nigra dermatological disease (bootstrap value = 0.79; Figure 8, nrSSU 18S) [50]. For the nrLSU (28S), a total of 28 accessions in addition to the current two isolates of *P. globosa* (DPS10 and MC769) showed an alignment length of 782 bp (incomplete sequence, partial at 3′ terminal). The number of identical sites was 573 bp (73.8%), while the un-gapped average length was 760 bp with GC% = 53.9%. The ML tree showed a monophyletic clustering of the families within order Capnodiales with the Extremaceae clade as root, followed by the Mycosphaerellaceae clade and the Teratosphaeriaceae clade. In the latter, the *P. globosa* DPS10 and MC769 isolates were highly clustered with two *P. globosa* accessions recorded as Capnodiales *incertae sedis* (bootstrap value = 0.99; Figure 8, nrLSU 28S).

Based on BT2, all retrieved sequences by BLAST without the intronic regions were discarded. A total of nine accessions and the current two isolates of *P. globosa* (DPS10 and MC769) showed an alignment length of 425 bp. The number of identical sites was 197 bp (48.2%), while the un-gapped average length was 385 bp with GC% = 55.6%. The isolates of *P. globosa* were included within the clades identified as order Capnodiales, family Teratosphaeriaceae (Figure 8, BT2). In RPB2, a total of 19 accessions and the current two isolates of *P. globosa* (DPS10 and MC769) showed an alignment length of 239 bp. The number of identical sites was 107 bp (44.8%), while the un-gapped average length was 239 bp with GC% = 53.1%. The isolates of *P. globosa* were included within the clades identified as order Capnodiales, family Teratosphaeriaceae with bootstrap value = 0.70 (Figure 8, RPB2).

## 4. Discussion

Black meristematic fungi or RIFs are remarkably extremotolerant microorganisms, frequently isolated from stone surfaces and habitats where minimal nutrients are available [51]. RIF survive under extreme conditions and grow to form micro-colonial fungal life forms (MCF) [52]. The selective pressure of such harsh conditions directed the evolution of their genomes to develop tolerant alleles through adaptive genetic variation and accumulation of beneficial mutations; the genetic pools of those organisms deviated significantly from sister fungal lineages to overcome the rock surface conditions (e.g., high UV radiation and temperatures) [53]. For instance, the highly concentrated black pigments in the MCF cell walls serve as a UV-protective substance (e.g., [54]).

This is the first report of *Pseudotaeniolina globosa* on Egyptian archaeological remains. *Pseudotaeniolina globosa* is one of the uncommonly recorded RIF; only a few records were previously reported. All the reported isolates are known as environmental saprobes, predominantly found in water-limited ecological niches. Except for a unique strain isolated from the aortic wall of a patient with an aortic aneurysm, its clinical significance has not been confirmed [45].

In the current work, the morphological measurements of the hyphal length and width on MEA media of DPS10 isolate (7.0–12.0 × 4.0–6.0 µm) were smaller than the MC769 (8.0–15.0 × 6.0–7.0 µm) [16]. However, the DPS10 produces single cells or asymmetrically septate cells after conidiogenesis, a unique character to *P. globosa* [16]. The current isolate is slimy and forms yeast-like colonies after 4–5 days of growth, which differentiate the *P. globosa* from a micro-morphologically similar species known as *Knufia petricola* (Wollenz. & de Hoog) Gorbushina & Gueidan (=*Sarcinomyces petricola* Wollenz. & de Hoog) [16,55,56]. Regardless of the morphological variation between both isolates, the molecular characterization of the DPS10 isolate confirms its identity and high similarity to the *P. globosa* MC769 isolate. However, under abiotic stress (pH, salinity, and temperature) the DPS10 showed tolerance to extreme conditions. 

The pH of a medium is a decisive factor for fungal presence and diversity; for example, the soil pH was the most significant factor correlated to the fungal community composition in Svalbard, High Arctic [57]. The DPS10 optimum growth was at 5.0–7.0 pH and it grew on a broader range from 3.0 to 9.0 pH compared to MC769 isolate, which grew well between 4.0–6.0 and scarcely at 7.0 pH. Thus, the ability of DPS10 to colonize different media with different pH ranges is worth further studying.

The DPS10 optimum growth was at 0.25 M (1.5%) NaCl and growth continued, although at lower rates, up to 5.2 M (30.0%) NaCl, while MC769 was strictly limited to 0.20 M (1.2%) NaCl supplemented MEA. Fungi are considered halophilic if they are isolated from sites with 1.7 M (~10.0%) NaCl, and halotolerant if sporadic isolates can grow in vitro at 3 M NaCl supplemented medium [58]. According to previous reports of model halotolerant fungi, *Debaryomyces hansenii* (Zopf) Lodder & Kreger-van Rij can grow in up to 3 M NaCl [59], *Hortaea werneckii* in up to 5 M NaCl [60], while *Wallemia ichthyophaga* Johan-Olsen thrives in 5.2 M NaCl, the latter of which is considered the most halophilic fungus; however, it is unable to grow without salt in its medium [61]. The MC769 isolate was previously described as halo-sensitive [16]. However, according to our results, the DPS10 isolate can be considered halotolerant [58]. At the intracellular Na^+^ and K^+^ ion accumulation level, it was shown that *D. hansenii* and *W. ichthyophaga* are Na^+^-intruder fungi and accumulate higher amounts of Na^+^ ions than *H. werneckii*, which excludes these ions from its cells [58]. Halophily is expressed in several groups of the same order, but not the closest at the taxonomic level [58]. *Hortaea werneckii* is the nearest phylogenetic neighbor to *P. globosa* and, most probably, follows the same mechanism. Alternatively, they are extreme xerotolerants whose growth on substrates is determined by water potential and not by the solute’s chemical nature [62]. 

DPS10 and MC769 showed optimum growth rate at 25 °C, but DPS10 had a broader range of growth temperatures (4–37 °C) than MC769 (10–30 °C). Additionally, DPS10 isolate was able to regrow after being exposed to 85 °C for 5 min while MC769 regrow only after being exposed to 80 °C for 5 min. Both *P. globosa* isolates are melanized, thus constitute a chemical ability to absorb and tolerate UV light [63]. Both isolates were tolerant to UV exposure up to 24 h; however, the growth rate was not affected up to 9 h in the DPS10 case compared to MC769, which was affected after 9 h of exposure to UV. Melanin serves as an extracellular electron-dense granular layer for black fungi and yeasts and forms an intracolonial matrix structure [52]. The results suggest that DPS10 is an extremotolerant *P. globosa* ecotype adapted to the harsh and extreme environments as arid and desert climate. It can survive pH changes, high salt concentrations, and tolerate high temperatures and UV radiation, demonstrating its potential as a persistent bio-deteriorating biological agent. Thus, further studies are required to understand its nature fully and develop counter strategies and conservation plans for one of Egypt’s oldest pyramids (i.e., the Djoser pyramid).

Within Ascomycota, the main orders with halophilic and halotolerant representatives are Capnodiales, Dothideales, and Eurotiales [58]. Among them, the Capnodiales have a xero-tolerant tendency, as they contain a large number of extremotolerant species that can grow as epilithic or crypto-endolithic species at high or low temperatures [6]. Most RIF possesses convergent morphological and physiological characteristics; however, many RIF subgroups are phylogenetically uncertain within the class Dothideomycetes [3]. According to the NCBI taxonomy database, the genus *Pseudotaeniolina* is uncertainly positioned within the order Capnodiales (*incertae sedis*) with no family assigned. When first reported, *P. globosa* was found clustered with species associated with low water availability (halophilic, epilithic, or epiphytic) and defined as RIF within the Dothideales but was poorly supported by the phylogenetic analysis using ITS sequences (>50%) [16]. In the current study, based on five molecular markers (ITS, SSU, LSU, BT2, and RPB2), *P. globosa* was clustered within the Teratosphaeriaceae family with bootstrap values of 0.70–0.98. 

Ruibal et al. [3] conducted a phylogenetic analysis using a multi-locus approach (nrSSU, nrLSU, and mtSSU) to resolve several taxonomic complexes of Dothideomycetes families. Among other families, the Teratosphaeriaceae was the most diverse and phylogenetically conflicting. The family Teratosphaeriaceae was represented as two separated clades, numbered as (1) and (2); however, the study did not include any *P. globosa* records. When we matched the species, the *P. globosa* isolates should be part of Teratosphaeriaceae group 1. When locus analyses conducted separately, the clustering was apparent at the ribosomal cistron sequences (ITS, SSU, and LSU) and resulted in a better resolution for the family compared to the closest one (Mycosphaerellaceae), especially the LSU. In contrast to other markers, the SSU phylogenetic signal was the only marker to show a paraphyletic clustering among the two proximate families, Mycosphaerellaceae and Teratosphaeriaceae; however, the taxonomical resolution within the Teratosphaeriaceae was not as structured as the ITS based phylogeny. The current study focused on the taxonomical family assignment of the *P. globosa* rather than on the taxonomical analysis of the order or on finding the best marker for such an assignment. However, many accessions were discarded and are missing in our analysis (e.g., RPB2) due to wrong taxonomic assignment or incomplete metadata about the accessions, which require urgent revision to avoid misleading phylogenetic signals. For instance, the *P. globosa* CCFEE 5734 RPB2 sequence (GenBank accessions KF310073) seems confused with *Extremus antarcticus* Quaedvl. & Crous isolate CCFEE 451 RPB2 sequence (GenBank accessions KF310085 [21]).

Future research will target the genomic signatures of the extremo-tolerance characters of the DPS10 isolate to accurately detect the transcriptional responses and metabolic suspension and reactivation under the fluctuation of water availability and its extreme ability to maintain viability in a high salinity medium. This will be achievable after acquiring additional *P. globosa* isolates and performing a more sophisticated comparison at genomic, transcriptomic, and metabolic levels using NGS-based approaches.

## Figures and Tables

**Table 1:** Multi-locus primers used for the identification of *Pseudotaeniolina globosa* from literature.

Locus	Primer (Direction)	Annealing (°C)	Reference
ITS	ITS1 (F)	50	[38]
	ITS4 (R)		
nrSSU	NS1 (F)	55	[38]
	NS3 (F)		
	NS5 (F)		
	NS7 (F)		
	NS24 (R)		
nrLSU	LSU1Fd (F)	52	[39]
	LR5 (R)		[40]
BT2	T1	52	[41]
	T22		
RPB2	fRPB2-5F	49	[42]
	fRPB2-5F+414R		[43]

**Table 2:** List of *Pseudotaeniolina globosa* available accession numbers in the GenBank database with an indication of the literature reference where they were published.

Isolate Code	Isolation Country	GenBank Accession Number					References
		ITS	nrSSU (18S)	nrLSU (28S)	BT2	RPB2	
CBS 303.84 *	Germany	AJ244268	-	-	-	-	[44]
CBS 110353	Germany	AJ244268 **	-	-	-	-	[45]
MC769 ***	Italy	AY128700	NG062782	MW367900	MW371112	MW371115	[16], current study
L10	Austria	HQ115663	-	-	-	-	[46]
ICP 1002	Austria	KC311489	-	-	-	-	[47]
CCFEE 5734	Italy	KF309976	-	KF310010	KF546758	KF310073	[21]
H19	Chili	KF578436	-	-	-		[48]
HF24	Austria	KR081416	-	-	-	-	[49]
DPS10	Egypt	MH396690	MH396869	MH396691	MW371113	MW371114	Current study

* Initially identified as *Trimmatostroma* sp; ** No sequences were reported; however, the cited reference was reported as similar to CBS 303.84; *** First report and description of the species; the isolate also recorded as CBS 109889^T^.

## Data Availability

Sequences were deposited in GenBank repository under the accession numbers detailed in Table 2.
